# Plasma Fibulin-1 Is Linked to Restrictive Filling of the Left Ventricle and to Mortality in Patients With Aortic Valve Stenosis

**DOI:** 10.1161/JAHA.112.003889

**Published:** 2012-12-19

**Authors:** Jordi S. Dahl, Jacob E. Møller, Lars Videbæk, Mikael K. Poulsen, Torsten R. Rudbæk, Patricia A. Pellikka, W. Scott Argraves, Lars Melholt Rasmussen

**Affiliations:** Department of Cardiology, Odense University Hospital, Denmark (J.S.D., L.V., M.K.P., T.R.R.); Department of Clinical Biochemistry and Pharmacology, Odense University Hospital, Denmark (L.M.R.); Department of Cardiology, Heart Center, Copenhagen University Hospital Rigshospitalet, Denmark (J.E.M.); Division of Cardiovascular Diseases, Mayo Clinic, Rochester, MN (P.A.P.); Department of Regenerative Medicine and Cell Biology, Medical University of South Carolina, Charleston, SC (W.S.A.)

**Keywords:** aortic valve stenosis, biomarkers, diastolic function, echocardiography

## Abstract

**Background:**

Plasma fibulin-1 levels have been associated with N-terminal pro–B-type natriuretic peptide levels and left atrial size and shown to be predictive of mortality in patients with diabetes. The mechanisms behind these connections are not fully understood but are probably related to its roles as an extracellular matrix protein in cardiovascular tissues.

**Methods and Results:**

One hundred twenty-five patients with severe aortic stenosis who were scheduled for aortic valve replacement (AVR) were evaluated with preoperative echocardiography and their plasma fibulin-1 levels were determined with ELISA. The cohort was followed for a median of 4 years after AVR. Increased restrictive left ventricular (LV) filling pattern was observed with increased plasma fibulin-1 levels (2% versus 29% versus 24% in low, middle, and high plasma fibulin-1 tertile groups, *P*=0.004). Likewise, reduced longitudinal systolic LV function (6.6±1.1 versus 6.1±1.3 versus 5.7±1.5 cm/s, *P*=0.05) and increased LV filling pressures was systolic velocity of the mitral annulus observed with increasing plasma fibulin-1 concentrations (ratio of early transmitral flow velocity to early diastolic flow velocity of the mitral annulus 13±4 versus 15±5 versus 16±6 in the fibulin-1 tertile groups, *P*=0.04).

**Conclusions:**

In patients with symptomatic severe aortic stenosis undergoing AVR, plasma fibulin-1 is associated with restrictive filling of the LV, decreased longitudinal systolic function of the LV, and increased LV filling pressures.

**Clinical Trial Registration:**

URL: http://www.clinicaltrial.gov with Identifier: NCT00294775

## Introduction

The cardiac consequences of aortic valve stenosis (AS) are characterized by chronic left ventricular (LV) pressure overload leading to LV hypertrophy and myocardial fibrosis, with both increasing myocardial stiffness. The consequence of increased myocardial stiffness is diastolic dysfunction, increased LV filling pressures, left atrial (LA) dilatation, shortness of breath, and increased risk of atrial fibrillation (AF).^1–4^ Increased myocardial stiffness may additionally prevent the remission of heart failure symptoms after aortic valve surgery^3,4^ worsening postoperative outcome.^4^ Markers of myocardial stiffness are thus warranted. A restrictive LV filling pattern assessed by echocardiography may identify patients with increased myocardial stiffness. Because LV filling pattern, however, also is affected by loading conditions and LV relaxation, increased myocardial stiffness may be present despite a nonrestrictive filling pattern.

Fibulin-1 is an extracellular matrix (ECM) and blood protein emerging as a biomarker in several cardiovascular diseases.^5,6^ During development, it is expressed in endocardial cushions, cardiac valves, and myocardium.^7–9^ In adults, it is also a prominent ECM component of cardiac valves, heart muscle, and blood vessels.^5,10^ We recently demonstrated an association between plasma fibulin-1 levels and the presence of cardiovascular disease and showed that it is predictive for all-cause and cardiovascular mortality in patients with diabetes.^5^ Fibulin-1 is upregulated in cardiomyopathy,^11^ and fibulin-1 blood levels are positively associated with plasma N-terminal pro–B-type natriuretic peptide (NT-proBNP) levels and LA size.^5,6^ In the present investigation, we evaluated the hypothesis that plasma fibulin-1 may be associated with LV diastolic impairment and increased LV filling pressures, which result in a poorer postoperative outcome in patients with AS.

## Methods

The present investigation is a substudy of a prospective single-center, randomized study to evaluate the effect of candesartan compared with conventional treatment on reverse remodeling in consecutive patients undergoing aortic valve replacement (AVR) for symptomatic AS. The study was registered with the National Board of Health and the Danish Data Protection Agency (http://ClinicalTrials.gov Identifier: NCT00294775) and was approved by the local ethical committee, and all patients gave written informed consent. The study design and effect of candesartan on regression of LV hypertrophy have previously been published.^12^ In brief, patients aged >18 years with symptomatic severe AS (estimated aortic valve area <1 cm^2^) scheduled for AVR at Odense University Hospital, Odense, Denmark, during February 2006 to April 2008 were enrolled. Patients with LV ejection fraction <40%, serum creatinine >220 μmol/L, previous aortic valve surgery, planned additional valve repair/replacement, infective endocarditis, predominant aortic valve regurgitation, or ongoing treatment with an angiotensin-converting enzyme inhibitor or an angiotensin receptor blocker were excluded. All patients undergoing AVR were enrolled in this study and divided among 3 groups according to fibulin-1 tertile.

### Echocardiography

All echocardiograms were performed by the same operator with a GE Vivid 5 ultrasound machine (GE Medical System, Horten, Norway) the day before surgery and were repeated 12 months after AVR. Echocardiograms were digitally stored and later analyzed in a blinded manner for all clinical data, survival data, and fibulin-1 levels. Aortic valve area was estimated with quantitative Doppler using the continuity equation. Peak flow velocity across the valve was determined in the window where the highest velocity could be recorded using continuous-wave Doppler with the cursor as parallel as possible with the flow across the valve. Peak transvalvular gradient was estimated using the modified Bernoulli equation. Finally, the peak systolic flow velocity in the outflow tract was estimated with pulsed-wave Doppler.^13^

As a measure of LV hypertrophy, LV mass was estimated according to the joint recommendations of the American Society of Echocardiography and European Association of Echocardiography using the Devereux formula: 0.8×(1.04 [LV internal diameter+posterior wall thickness+interventricular septal thickness]^3^ − [LV internal diameter]^3^)+0.6 g. Diastolic LV wall thickness and dimensions were estimated from the average of 3 consecutive frozen 2-dimensional images obtained in the parasternal long axis.^14^ In men and women, LV mass index (LVMi) >116 g/m^2^ and >100 g/m^2^, respectively, was considered indicative of LV hypertrophy.^15^ Relative wall thickness was calculated using the formula 2×posterior wall thickness/LV internal diameter in diastole.^16^

LV ejection fraction was estimated using the Simpson biplane method. Longitudinal LV systolic function was assessed using peak systolic mitral annular motion assessed with tissue Doppler imaging with the Doppler sample volume placed in the septal and lateral mitral valve annulus.

Mitral inflow was assessed in the apical 4-chamber view using pulsed-wave Doppler with the sample volume paced at the tips of mitral leaflets during diastole. From the mitral inflow profile, the E- and A-wave peak velocities and deceleration time were measured. Doppler tissue imaging of the mitral annulus was used in the aforementioned sampling sites to measure the early diastolic e′ velocity from each site. The ratio of early transmitral flow velocity to early diastolic flow velocity of the mitral annulus (E/e′ ratio) was used as a noninvasive marker of LV filling pressures.^17^ The diastolic and restrictive filling patterns were categorized according to European Association of Echocardiography guidelines.^18^

LA volume was assessed using the area–length method^15^ from the apical 4- and 2-chamber views. Measurements were obtained in end-systole from the frame preceding mitral valve opening, and the volume was indexed for body surface area.

### Biochemical Analysis

Blood samples were collected immediately after the echocardiogram, after the subject had been resting recumbent for at least 30 minutes. Samples were collected in EDTA-containing tubes. The tubes were then centrifuged, and plasma samples were stored at −80°C for later analysis. A sandwich immunoassay for fibulin-1 was used for measuring fibulin-1 levels.^5,19^ NT-proBNP and creatinine levels were analyzed with a Modular Analytics P device (Roche Diagnostics, Indianapolis, IN, USA).

### Assessment of Atrial Fibrillation

Patients were examined 1, 3, 6, 9, and 12 months after AVR and underwent a 12-lead resting ECG for 2 minutes in the supine position. In addition, a 24-hour Holter electrocardiographic recording was performed 3 months after surgery, and a 48-hour Holter was performed at 12 months. These recordings were performed with a Reynolds Medical Tracker 3 and a Pathfinder 700 (Reynolds Medical Limited, England) for analysis. All ECGs were interpreted by the same experienced cardiologist, who was blinded to treatment allocation and fibulin-1 levels. An episode of irregular heart rhythm with no definite p waves and with a duration >30 seconds was considered to be AF according to guidelines.^20^ Episodes of AF occurring within 30 days of AVR were considered to be postoperative AF and were not recorded as an end point.

Patients with known AF before AVR were excluded in analyses estimating the association of fibulin-1 with the development of AF.

### Clinical Examination and Follow-up

All patients had coronary angiography performed before AVR and underwent a thorough clinical examination on the day before AVR. Arteriosclerosis was defined as previous history of cerebral stroke, aortic disease, peripheral arterial disease, or coronary heart disease or if arteriosclerosis was present on the preoperative coronary angiogram. In addition operative risk was estimated using the European System for Cardiac Operative Risk Evaluation (EuroSCORE).^21^

By July 2011, data on outcomes were collected from the Danish Personnel Register (survival status) and from discharge notes available in the Danish admission registry. In case of ambiguous information, local hospitals were contacted and detailed patient charts were reviewed.

The main end point for this study was cardiovascular mortality; the secondary end point was AF.

### Statistics

Data are presented as mean±SD or number and percentages. Differences between groups were tested by ANOVA; categorical variables were tested by Fisher exact test. Due to a nongaussian distribution, NT-proBNP was logarithm transformed. The association of fibulin-1 with arteriosclerosis and restrictive filling was tested by multivariate ANOVA. Mortality and event rates were calculated using the product–limit method and plotted according to the Kaplan–Meier method; death rates were compared using the log-rank test. Further estimation of risk was performed using Cox proportional hazard models. In addition to hazard ratios, adjusted hazard ratios for 1 SD were calculated, dividing the variable by the SD of the measure. We used DAGitty, a web-based application based on the directed acyclic graph theory, to model the causal relationship between fibulin-1 and cardiovascular mortality and any confounders.^22^ This DAG program was used to identify sets of confounders that together fully adjust for confounding in multivariable modeling.^23^ Several of these sets of confounders were identified (Figure S1). The set containing age, history of diabetes, AS severity, and LVMi was used for multivariable analysis.

The assumptions (proportional hazard assumption, linearity of continuous variables, and lack of interaction) were tested and found to be valid.

A *P* value <0.05 was considered significant. STATA/SE 9.0 (StataCorp LP, College Station, TX, USA) software was used for statistical analysis.

## Results

Table 1 provides baseline clinical data for 125 patients stratified according to plasma fibulin-1 level, which was 89±33 μg/mL in the complete cohort. Fibulin-1 level was 57±9 μg/mL in the first tertile, 85±9 μg/mL in the second tertile, and 125±27 μg/mL in the third tertile. Age and history of diabetes, coronary heart disease, peripheral artery disease, AF, and stroke were similar between groups. There was a predominance of women in the 2 upper tertiles (19% versus 48% versus 54%, *P*=0.01). There was a trend toward an increased EuroSCORE result in patients with increased plasma fibulin-1 levels (5.3±1.9 versus 5.9±1.8 versus 6.1±2.1, respectively; *P*=0.13).

**Table 1.** Characteristics of Patients

	Fibulin-1 First Tertile (n=42)	Fibulin-1 Second Tertile (n=42)	Fibulin-1 Third Tertile (n=41)	*P*
Age, y	71±8	72±11	74±8	0.36

Male sex	34 (81)	22 (52)	23 (56)	0.01

Hypertension	22 (52)	15 (36)	16 (39)	0.26

Arteriosclerosis	21 (50)	17 (40)	19 (46)	0.68

Diabetes mellitus	5 (12)	6 (14)	8 (20)	0.62

AF	4 (5)	5 (12)	10 (24)	0.13

Ischemic heart disease	9 (21)	7 (17)	7 (17)	0.82

Peripheral artery disease	5 (12)	2 (5)	4 (10)	0.50

Stroke	1 (2)	3 (7)	4 (10)	0.38

EuroSCORE	5.3±1.9	5.9±1.8	6.1±2.1	0.13

Logistic EuroSCORE	4.6±3.1	5.6±3.3	6.3±5.3	0.16

Diuretic therapy	17 (40)	11 (26)	16 (39)	0.32

β-Blocker therapy	8 (19)	9 (21)	11 (27)	0.69

CCB therapy	11 (26)	7 (17)	7 (17)	0.47

Candesartan	19 (45)	21 (50)	23 (56)	0.61

Symptoms				

6-min walk test, m	348±126	338±123	347±128	0.94

NYHA functional class	2.2±0.7	2.0±0.7	2.1±0.7	0.30

NYHA functional class 1/2/3/4	6/20/16/0	9/24/9/0	8/21/11/1	0.52

Creatinine, μmol/L	98±21	97±19	105±24	0.22

Plasma fibulin-1, μg/mL	57±9	85±9	125±27	

Data are n (%) or mean±SD or n. AF indicates atrial fibrillation; CCB, calcium channel blocker; NYHA, New York Heart Association.

AVR was performed in all patients. Coronary artery bypass grafting was performed in 37 (30%) patients with no difference between groups (Table 1); complete revascularization was achieved in all patients. No difference in the size or type of aortic valve prosthesis was seen between groups; additionally, the use of β-blockers, calcium-channel blockers, angiotensin-converting enzyme inhibitors, and diuretics was equal between groups preoperatively. Randomization to candesartan treatment was similar between groups (Table 1). A poor but significant correlation was seen between creatinine and fibulin-1 levels (*r*=0.18, *P*=0.042; data not shown). Creatinine levels were, however, similar between groups (98±21 versus 97±19 versus 105±24 μmol/L, respectively; *P*=0.22) (Table 1). Table 2 shows echocardiographic data distributed among groups.

**Table 2.** Preoperative Echocardiographic Data

	Fibulin-1 First Tertile (n=42)	Fibulin-1 Second Tertile (n=42)	Fibulin-1 Third Tertile (n=41)	*P*
AVA, cm^2^	0.9±0.3	0.7±0.2	0.8±0.3	0.07

AV velocity, m/s	3.7±0.7	4.0±0.7	4.0±0.9	0.17

LVMi, g/m^2^	121±32	128±41	144±45	0.03

IVS, mm	12±2	13±2	14±3	0.002

LV posterior wall thickness, mm	13±2	13±2	14±2	0.11

LV hypertrophy	27 (64)	28 (67)	32 (78)	0.35

LAVi, mL/m^2^	45±19	46±17	55±18	0.04

LVEDD, mm	46±6	44±7	45±6	0.31

Mitral E velocity, m/s	0.7±0.2	0.8±0.2	0.8±0.3	0.14

Mitral A velocity, m/s	1.0±0.3	0.9±0.3	1.0±0.3	0.37

Mitral DT, ms	219±60	185±52	190±59	0.01

Restrictive filling pattern	1 (2)	12 (29)	10 (24)	0.004

Diastolic function				

0/1/2/3	2/22/17/1	1/9/20/12	3/14/13/10	0.02

e′_sep_, cm/s	5.7±1.5	5.9±1.8	5.7±1.7	0.92

E/e′_sep_	13±4	15±5	16±6	0.04

Systolic function				

EF, %	54±7	54±9	55±6	0.91

S′_sep_, cm/s	6.6±1.1	6.1±1.3	5.7±1.5	0.05

log NTproBNP	5.8±1.1	6.2±1.4	6.7±1.3	0.008

Data are n (%) or mean±SD or n. AVA indicates aortic valve area; AV, aortic valve; IVS, interventricular septal thickness; LAVi, left atrial volume index; LVEDD, left ventricular end diastolic diameter; DT, deceleration time; EF, ejection fraction; NT-proBNP, N-terminal pro–B-type natriuretic peptide; E'_sep_, early diastolic velocity of the septal mitral annulus; S'_sep_, systolic velocity of the septal mitral annulus.

A trend toward decreased aortic valve area was seen in patients with increased plasma fibulin-1 levels (0.9±0.3 versus 0.7±0.2 versus 0.8±0.3 cm^2^, respectively; *P*=0.07). Plasma fibulin-1 correlated positively with indices of LV hypertrophy (LVMi: *r*=0.19, *P*=0.034; interventricular septal thickness: *r*=0.21, *P*=0.02). The presence of LV hypertrophy was, however, not significantly different between groups (64% versus 67% versus 78%, respectively; *P*=0.35). Increased plasma fibulin-1 levels were associated with increased occurrence of LV restrictive filling, elevated filling pressures, and increased LA volume index (45±19 versus 46±17 versus 55±18 mL/m^2^, respectively; *P*=0.04). In addition, plasma fibulin-1 levels were associated with reduced longitudinal systolic LV function measured as s′_sep_ (6.6±1.1 versus 6.1±1.3 versus 5.7±1.5 cm/s, respectively; *P*=0.05). Plasma fibulin-1 was increased in patients with LV restrictive filling independent of the presence of arteriosclerosis (87±31 versus 100±26 μg/mL in patients without arteriosclerosis and 84±34 versus 123±35 μg/mL in patients with arteriosclerosis; multivariate ANOVA *P*_restrictive filling_=0.009, *P*_arteriosclerosis_=0.94).

LVMi regression during follow-up was not significantly different between groups (−14±25 versus −20±36 versus −31±42 g/m^2^, respectively; *P*=0.15) (Table 3), although LVMi was similar between groups (113±35 versus 107±29 versus 112±29 g/m^2^, respectively; *P*=NS) at 12 months after surgery. Although ejection fraction at 12 months after AVR was similar between groups, s′_sep_ remained reduced in patients with increased preoperative fibulin-1 levels (7.1±1.5 versus 6.3±1.3 versus 6.1±1.4 cm/s, respectively; *P*=0.007).

**Table 3.** Echocardiographic Data 12 Months After AVR

	Fibulin-1 First Tertile (n=35)	Fibulin-1 Second Tertile (n=35)	Fibulin-1 Third Tertile (n=33)	*P*
LVMi, g/m^2^	113±35	107±29	112±29	0.72

IVS (mm)	12±3	12±2	12±2	0.91

LV posterior wall thickness, mm	12±2	12±2	12±2	0.39

LAVi, mL/m^2^	47±16	42±14	52±18	0.04

LVEDD, mm	45±6	44±6	45±5	0.75

Mitral E velocity, m/s	0.9±0.3	0.9±0.3	0.9±0.3	0.93

Mitral A velocity, m/s	1.0±0.2	0.9±0.2	1.0±0.3	0.24

Mitral DT, ms	218±45	198±61	183±54	0.04

e′_sep_, cm/s	6.3±1.6	6.1±1.4	5.9±1.9	0.65

E/e′_sep_	15±7	15±7	17±8	0.71

Systolic function				

EF (%)	53±7	51±7	54±9	0.38

S′_sep_ (cm/s)	7.1±1.5	6.2±1.3	6.1±1.4	0.007

log NT-proBNP	5.9±1.2	5.8±1.1	6.1±1.0	0.51

Plasma fibulin-1, μg/mL	63±13	89±17	121±33	<0.001

Difference during follow-up				

LVMi, g/m^2^	−14±25	−20±36	−31±42	0.15

EF, %	−1±7	−2±11	−1±11	0.88

S′_sep_, cm/s	1.0±1.2	0.4±1.6	0.6±1.5	0.31

LAVi, mL/m^2^	−0.2±11	−3.3±12	−2.4±14	0.61

Plasma fibulin-1, μg/mL	6.6±11.3	3.8±19	0.6±27	0.51

Data are n (%) or mean±SD. AVR indicates aortic valve replacement; LVMi, left ventricular mass index; IVS, interventricular septal thickness; LV, left ventricular; LAVi, left atrial volume index; LVEDD, left ventricular end diastolic diameter; DT, deceleration time; EF, ejection fraction; NTproBNP, N-terminal pro–B-type natriuretic peptide E'_sep_, early diastolic velocity of the septal mitral annulus; S'_sep_, systolic velocity of the septal mitral annulus.

### Atrial Fibrillation

Twenty-nine patients developed AF during the first postoperative year: 7 in the first group, 9 in the second group, and 13 in the third group. In a univariable Cox regression model, plasma fibulin-1, LA volume index, age, plasma NT-proBNP, and LVMi were associated with the development of AF. After adjustment for age and LAVi, only plasma fibulin-1 remained significantly associated with AF development (Table 4).

**Table 4.** Univariable Predictors of AF

	Univariable			Univariable*		
		
	HR (95% CI)	HR_prSD_	*P*	HR	HR_prSD_	*P*
Fibulin-1	1.01 (1.00 to 1.02)	1.49	0.01	1.01	1.41	0.04

Fibulin-1 tertiles						
					
First	1					
					
Second	1.4 (0.5 to 3.8)		0.50			
					
Third	2.2 (0.9 to 5.5)		0.09			
			
LVEF, %	0.99 (0.95 to 1.05)	0.99	0.99			
			
Age, /y	1.06 (1.01 to 1.12)	1.70	0.02			
			
EuroSCORE	1.17 (0.97 to 1.41)	1.36	0.10			
			
Diabetes mellitus	1.00 (0.34 to 2.9)		0.99			
			
LAVi, mL/m^2^	1.03 (1.01 to 1.05)	1.69	0.001			

LVMi, g/m^2^	1.01 (1.00 to 1.02)	1.46	0.014	1.00	1.03	0.87

E/e′_avg_	1.04 (0.97 to 1.11)	1.23	0.26			

NT-proBNP	1.00 (1.00 to 1.00)	1.42	0.001	1.00	1.19	0.25

LVEDD, cm	1.18 (0.65 to 2.1)	1.11	0.59			

S′_sep_, cm/s	0.81 (0.63 to 1.04)	0.73	0.10	0.69	0.98	0.93

AF indicates atrial fibrillation; HR, hazard ratio; HR_prSD_, hazard ratio per SD; LVEF, left ventricular ejection fraction; LAVi, left atrial volume index; LVEDD, left ventricular end-diastolic diameter; NT-proBNP, N-terminal pro–B-type natriuretic peptide; S'_sep_, systolic velocity of the septal mitral annulus.

* Adjusted for age and LAVi.

### Clinical Outcome

The mean follow-up duration after AVR in the total cohort was 3.8±1.5 years (median 4.0 years). Survival status was available for all patients. Overall, there were 29 deaths: 8 in the first tertile, 8 in the second tertile, and 13 in the third tertile. The cause of 4 deaths was not a cardiac condition (n=2, cancer; n=1, infectious disease; n=1, subarachnoid hemorrhage), and 25 patients had a cardiac cause of death (n=15, sudden cardiac death; n=7, postoperative death; n=2, congestive heart failure; n=1, aortic aneurysm).

**Figure 1.**
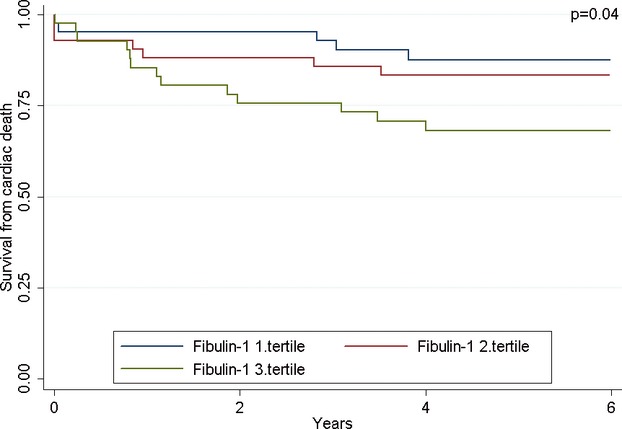
Kaplan–Meier plot showing survival free of cardiac death after AVR on fibulin-1 tertile. LV indicates left ventricular; EF, ejection fraction; CV, cardiovascular; NT-proBNP, N-terminal pro–B-type natriuretic peptide; AVR, aortic valve replacement.

Overall mortality and cardiac mortality were significantly increased in patients with increased plasma fibulin-1 levels (estimated 5-year cardiac mortality rate: first tertile 13% [n=5], second tertile 17% [n=7], third tertile 32% [n=13], *P*=0.04, Figure 1).

In a univariable Cox regression analysis, fibulin-1, LA volume index, LVMi, and s′_sep_ were associated with cardiac survival (Table 5). No effect modification was demonstrated by sex, age, LA volume, LVMI, or diabetes on the association between fibulin-1 and cardiovascular mortality.

**Table 5.** Univariable and Multivariable Predictors of Cardiac Death

	Univariable			Univariable*			Multivariable		
			
	HR (95% CI)	HR_prSD_	*P*	HR	HR_prSD_	*P*	HR	HR_prSD_	*P*
Fibulin-1	1.01 (1.00 to 1.03)	1.62	0.004	1.01	1.56	0.009	1.01	1.41	0.04

Fibulin-1 tertiles									
								
First	1			1					
							
Second	1.44 (0.5 to 4.5)	0.54		1.46		0.53			
							
Third	2.89 (1.0 to 8.1)	0.04		2.69		0.04			
			
LVEF, %	0.97 (0.92 to 1.02)	0.77	0.99	0.97	0.80	0.27			
			
Female sex	1.26 (0.6 to 2.8)		0.58						
						
Age, /y	1.05 (0.99 to 1.10)	1.53	0.07				1.04	1.42	0.15
				
EuroSCORE	1.20 (0.99 to 1.45)	1.46	0.06						

Diabetes	3.2 (1.4 to 7.3)		0.007	3.4		0.005	3.3		0.01
				
IHD	0.80 (0.3 to 2.3)		0.69	0.82		0.88			
							
Hypertension	0.78 (0.2 to 1.8)		0.54	0.72		0.43			

AVA	1.85 (0.5 to 7.1)	1.18	0.37	2.43	1.28	0.20	3.1	1.36	0.07

LAVi	1.02 (1.00 to 1.04)	1.47	0.03	1.02	1.36	0.10			

LVMi	1.01 (1.00 to 1.02)	1.40	0.047	1.01	1.29	0.16	1.01	1.37	0.12

E/e′_avg_	1.07 (1.00 to 1.15)	1.32	0.06	1.05	1.25	0.29			
			
NT-proBNP	1.00 (1.00 to 1.00)	1.11	0.50	1.00	1.04	0.83			
			
LVEDD	0.96 (0.51 to 1.8)	0.95	0.89	0.89	0.93	0.75			
			
S′_sep_	0.74 (0.56 to 0.98)	0.64	0.04	0.78	0.69	0.11			

HR indicates hazard ratio; HR_prSD_, hazard ratio per SD; LVEF, left ventricular ejection fraction; IHD, ischemic heart disease; AVA, aortic valve area; LAVi, left atrial volume index; LVMi, left ventricular mass index; LVEDD, left ventricular end-diastolic diameter; NT-proBNP, N-terminal pro–B-type natriuretic peptide.

* Adjusted for sex and age.

In a multivariable Cox model including the predefined variables of age, LA volume, and diabetes, only fibulin-1 and a history of diabetes mellitus were associated with cardiac mortality (Table 5).

## Discussion

The main finding in our study is that increased levels of plasma fibulin-1 associate with restrictive LV filling and increased LV filling pressures in patients with AS. Second, plasma fibulin-1 appeared to be associated with the development of AF and long-term postoperative cardiovascular mortality when adjusted for other factors in the study patients. These observations add to the emerging evidence that plasma fibulin-1 levels are reflective of cardiovascular disease, including findings showing that plasma fibulin-1 levels are associated with LA size and blood levels of NT-proBNP^5^ in patients with diabetes mellitus.

Fibulin-1 is an ECM protein^24,25^ prominently expressed during the development in endocardial cushions, cardiac valves, outflow track, blood vessels, and the ventricular myocardium. Indeed, mice deficient in fibulin-1 develop outflow tract and ventricular myocardial abnormalities and defects in peripheral blood vessels that are associated with hemorrhage.^9,26^ Fibulin-1 is also a prominent constituent of the cardiovascular system, where it is expressed in cardiac valves, cardiac muscle, and blood vessels, including the aorta and coronary and carotid arteries.^5,10^ Despite extensive expression of fibulin-1 in cardiovascular tissues, often in relation to elastin-containing fibers and elastic laminae,^5,10^ little is known about its function. The idea that fibulin-1 may reflect accumulation of ECM in fibrotic myocardium and correlations found in the present study fit with a recent Brazilian study demonstrating that myocardial fibrosis is a prognostic marker for postoperative outcome.^3,4^ The main constituents of the myocardial matrix are type I and, to a lesser extent, type III collagen. Interestingly, the myocardium also contains small amounts of elastin, which may play an important beneficial functional role, particularly in relation to the preservation of diastolic function.^27,28^ Whether our findings of changes in the elastin-associated matrix molecule fibulin-1 in relation to restrictive filling of the LV should be interpreted in this context remains to be seen.

Fibulin-1 circulates in relatively high concentrations in plasma^5,19^; however, the source of fibulin-1 in the blood is not known. Considering that myocardial fibulin-1 mRNA levels have been shown to increase in an animal model of cardiomyopathy^11^ and in a human model of the arterial wall in diabetes,^5^ it is possible that different cardiovascular pathologic conditions may augment tissue fibulin-1 to be released into the blood. Because fibulin-1 is expressed in valves,^10^ it is possible that fibrotic valves could also contribute fibulin-1 to the blood.

As stated earlier, our previous study established a link between plasma fibulin-1 and LA dilatation and plasma NT-proBNP concentrations in diabetic patients. We suggested that these associations could be secondary to increased arterial stiffness, because we observed increased arterial fibulin-1 concentrations in the arterial ECM in individuals with type 2 diabetes and because there was a correlation between plasma fibulin-1 and carotid compliance.^5^ Increased vessel stiffness could lead to premature return of reflected pulse waves in late systole,^29,30^ causing abnormal LV relaxation and impaired LV filling. However, filling of the LV is affected by several important myocardial factors, in addition to myocardial relaxation, atrial pressure, and LV compliance. AS is often accompanied by LV remodeling leading to altered LV compliance but is also associated with arteriosclerosis and arterial stiffness. In the present study, significant differences in fibulin-1 levels between patients with or without known arteriosclerosis were not established, but still we find associations between fibulin-1 and measures of restrictive filling pattern. This filling pattern with rapid pressure equalization (reduced deceleration time) is only seen in the presence of a stiff myocardium and elevated filling pressures, as opposed to impaired relaxation pattern with a long deceleration time, which is predominant among patients with arterial stiffness. The observed association between plasma fibulin-1 and restrictive LV filling pattern is therefore compatible with the idea that plasma fibulin-1 directly reflects the degree of myocardial stiffness. Decreased LV compliance and increased LA pressures will eventually dilate the LA, increasing the risk of AF. In line with this notion, we find that plasma fibulin-1 levels predict AF when adjusted for LA size, suggesting an effect not merely related to changes in hemodynamic parameters. Interestingly, novel findings of Garcia et al^31^ recently demonstrated that fibulin-1 levels are significantly altered in atrial tissue of patients with AF, although decreased levels were observed in diseased tissue. It is known that atrial fibrosis is associated with AF independent of filling hemodynamics^32^; it is possible that fibulin-1 plays a central role in the development of aberrant ECM in the atrial tissue of individuals with AF. Weidemann and colleagues recently demonstrated an association between increased fibrosis assessed by MRI and reduced longitudinal systolic function measured with tissue Doppler in AS.^3^ It is therefore intriguing that we observed reduced s′ velocities in patients with increased fibulin-1 levels, compatible with the idea that plasma fibulin-1 could reflect myocardial fibrosis.

### Study Limitations

Our sample size is relatively small, with few events, which makes our models unstable because confounding may be present; the small sample size does not allow control for potential confounders. Larger studies are therefore warranted. The entry criterion for the study was symptomatic AS referred for AVR. Future studies should also include asymptomatic patients to clarify whether our findings apply to a general population with AS.

LV structure was assessed by echocardiography, and no histologic examinations were performed; thus, we can only speculate on the degree of myocardial and valvular pathologic conditions. No direct hemodynamic measurements of LV end-diastolic or LA pressure were performed. However, E/e′ is accepted as a well-validated surrogate in a wide range of patients with cardiac disease including AS.^17^

### Clinical Implication

The present study demonstrates that preoperative plasma fibulin-1 levels in patients with AS are associated with restrictive filling of the LV, decreased longitudinal systolic function of the LV, and increased LV filling pressures. Moreover, plasma fibulin-1 levels are associated with AF and long-term postoperative mortality when adjusted for other factors in these patients. Thus, plasma fibulin-1 may be a new candidate marker to be used in clinical settings and research investigations to assess myocardial stiffness.
